# Heterogeneity of Estrogen Receptor Expression in Circulating Tumor Cells from Metastatic Breast Cancer Patients

**DOI:** 10.1371/journal.pone.0075038

**Published:** 2013-09-18

**Authors:** Anna Babayan, Juliane Hannemann, Julia Spötter, Volkmar Müller, Klaus Pantel, Simon A. Joosse

**Affiliations:** 1 Department of Tumor Biology, University Medical Center Hamburg-Eppendorf, Hamburg, Germany; 2 Department of Gynecology, University Medical Center Hamburg-Eppendorf, Hamburg, Germany; Health Canada and University of Ottawa, Canada

## Abstract

**Background:**

Endocrine treatment is the most preferable systemic treatment in metastatic breast cancer patients that have had an estrogen receptor (ER) positive primary tumor or metastatic lesions, however, approximately 20% of these patients do not benefit from the therapy and demonstrate further metastatic progress. One reason for failure of endocrine therapy might be the heterogeneity of ER expression in tumor cells spreading from the primary tumor to distant sites which is reflected in detectable circulating tumor cells (CTCs).

**Methods:**

A sensitive and specific staining protocol for ER, keratin 8/18/19, CD45 was established. Peripheral blood from 35 metastatic breast cancer patients with ER-positive primary tumors was tested for the presence of CTCs. Keratin 8/18/19 and DAPI positive but CD45 negative cells were classified as CTCs and evaluated for ER staining. Subsequently, eight individual CTCs from four index patients (2 CTCs per patient) were isolated and underwent whole genome amplification and *ESR1* gene mutation analysis.

**Results:**

CTCs were detected in blood of 16 from 35 analyzed patients (46%), with a median of 3 CTCs/7.5 ml. In total, ER-negative CTCs were detected in 11/16 (69%) of the CTC positive cases, including blood samples with only ER-negative CTCs (19%) and samples with both ER-positive and ER-negative CTCs (50%). No correlation was found between the intensity and/or percentage of ER staining in the primary tumor with the number and ER status of CTCs of the same patient. *ESR1* gene mutations were not found.

**Conclusion:**

CTCs frequently lack ER expression in metastatic breast cancer patients with ER-positive primary tumors and show a considerable intra-patient heterogeneity, which may reflect a mechanism to escape endocrine therapy. Provided single cell analysis did not support a role of *ESR1* mutations in this process.

## Introduction

Breast cancer is the most common malignancy among women, accounting for approximately 23% of all cancer cases. Furthermore, breast cancer represents the most frequent cause of cancer related death in women worldwide [1]. On the molecular level, breast cancer is a heterogeneous disease and several molecular subtypes have been described based on gene expression profiles and immunohistochemistry [2]–[4] that might be explained by their cell of origin [5]. The most common subtype is the luminal A type, presenting up to 50–60% of all breast cancer cases [2], [6]. These tumors are characterized by high estrogen receptor alpha (ER) expression and are - due to their low proliferation rate - associated with a relatively good prognosis [6], [7]. The luminal B subtype represents 10–20% of all breast tumors and is characterized by a mixed expression of ERα, PR, and/or ERBB2. It is often represented by an more aggressive phenotype of breast cancer with higher tumor grade [8].

A breast tumor’s ER expression is normally assessed by immunohistochemistry and the definition of ER “positive” status is based on the presence of 1% or more ER positive tumor cells [9]. Expression of ER often mediates sensitivity of these tumors to hormonal treatment with either selective estrogen receptor modulators, such as tamoxifen, or aromatase inhibitors. Although the therapeutic efficacy of endocrine treatment for women with ERα–positive primary or metastatic disease has been clearly demonstrated [10], [11], failure of therapy is observed in 20–25% of patients [12], [13]. More importantly, these patients demonstrate endocrine therapy “experienced progression” [12], meaning either de novo or acquired resistance to endocrine therapy. Resistance to endocrine therapy has been correlated to both ER-dependent [14] and ER-independent reasons [13]. To ER-dependent mechanisms belong genetic and/or epigenetic changes of the ERα gene, causing either lack of ERα protein expression or a dysfunctional ERα pathway [14] (*e.g.*, due to *ESR1* promoter hypermethylation, expression of truncated isoforms of ERα, post-translational modifications, and other genetic changes of ERα [15]). ER-independent ways of acquired endocrine resistance include alteration in cell cycle and cell survival signaling molecules, activation of escape pathways [13]. Failure of systemic therapy may eventually lead to outgrowth of metastases in distant organs and cancer-related death.

The putative precursors of distant metastases are circulating tumor cells (CTCs). These cells have detached from the primary tumor, circulate in the bloodstream, and may finally extravasate to metastasize [16]–[20]. CTC analysis hold great promise to be used to monitor adjuvant therapy efficacy, as a prognostic marker, for early detection of minimal residual disease [19], [21], and as a predictive marker for individualized cancer treatment [22]. Easy accessibility and possibility of sequential blood analyses make CTC analysis a promising new blood-based biomarker [22]–[25].

Several techniques have been developed for the enrichment and detection of CTCs, including assays based on cell size, immunological properties, and physical properties of the tumor cells (reviewed in [22], [23]). CTCs might be discriminated from leukocytes with high precision using their origin specific makers. CTCs, originating from carcinomas, normally express epithelial markers such as EpCAM (epithelial cell adhesion molecule) and keratins, on the other hand, CD45 molecules, also known as leukocyte common antigen, are expressed on the surface of white blood cells only (reviewed in [26]). Thus, the use of differently labeled antibodies against these specific markers allows to distinguish between CTCs and leukocytes.

Recently it was shown that the presence of CTCs after completion of adjuvant therapy is a predictor of metastatic relapse and poor survival [19], [27]–[30]. Additionally, prognostic information provided by CTCs might not be limited to the amount of CTCs only. CTCs might reflect the primary tumor’s biology, including intratumoral heterogeneity. Breast tumors are considered being ERα positive if 1% of the cells show nuclear reactivity of any intensity by immunohistochemical investigation [9]. Therefore, CTCs arising from primary ERα-positive breast tumors are not necessarily expected to be ERα-positive. ER-negative CTCs might originate from ER-negative cells of ER-mosaic primary tumor [31], or ER-negative clones might be selected and get growth superiority under the pressure of anti-ER therapy [32]. Appearance of genomic or epigenomic aberrations might also result in the appearance of ER-negative CTCs [33]. Since endocrine treatment is dependent on the hormone receptor status and targets ERα-positive cancer cells only, CTC heterogeneity might be one reason for treatment failure and metastasis development in patients with ERα-positive tumors.

Both ER-positive and ER-negative cells can be identified in therapy naïve primary tumors. Moreover, it was shown that ER status changes from positive to negative in 2.5–17.0% of the cases after therapy [34] and changing is possible in both directions [35]. In metastatic breast cancer, a change of ER status in comparison to the primary tumor was found in 17% of the cases [36]. Moreover, it is proposed, that the change from ER-positivity to ER-negativity might be one of the mechanisms to evade hormonal treatment (reviewed in [13], [33]).

Recent studies could show that divergence of hormone receptor status between primary tumor and CTCs is not a rare event [37]–[39]. However, in all of these studies polymerase chain reaction (PCR) assays were conducted based on the measurement of mRNA expression levels in a total CTC population. Using such an approach, intra-patient heterogeneity between single CTCs cannot be seen. Investigating ERα status of single CTCs might shed light on the cause of endocrine therapy resistance in individuals and ultimately lead to treatment optimization.

Therefore, in this study we present a highly sensitive approach to detect CTCs and simultaneously investigate their ER expression in blood samples from 35 metastatic breast cancer patients with ER-positive luminal primary tumors. Moreover, our method allows further genetic analyses of these single CTCs which is not possible in most of the commonly used immunostaining protocols due to fixation and crosslinking of the DNA.

## Materials and Methods

### Cell culture

Two ER-negative (BT-20 and MDA-MB-231) and two ER-positive (BT474 and MCF7) breast cancer cell lines were used. All cell lines were acquired from American Type Culture Collection and cultured under the prescribed conditions: MCF7, BT-20, and MDA-MB-231 cells were cultivated in DMEM (catalog no. E15-011, PAA Laboratories) at 37°C at 10% CO_2_; BT474 cells were cultivated in RPMI (catalog no. E15-039, PAA Laboratories) at 37°C at 5% CO_2._ Both media were supplemented with 10% fetal bovine serum (catalog no. E15-105, PAA Laboratories). Cells were grown in a 75 cm^2^ flask until confluency was reached. Cells were harvested using trypsin/EDTA (catalog no. R001100; Gibco), washed with PBS (catalog no. 14190-094; Gibco), and resuspended in 1×PBS for either spiking experiments or cytospin preparation for direct staining.

### Patients and blood sampling

Thirty five metastatic breast cancer patients with ER-positive primary tumors were included into the study. Average time between primary diagnosis and diagnosis of metastases was 7.2 years (range: 0.5–17.0 years). Median follow up was 13.1 months (range 1–30 month) starting from the time point of blood analysis. Patient details are described in Table S1. Patients were treated for metastatic breast cancer at the University Medical Center Hamburg-Eppendorf and received therapy, according to international guidelines.

Blood from five apparently healthy women of age 25–35 years was included into the study to function as negative control for the establishment of our protocol. All patients and healthy volunteers gave written informed consent to be included into the study. The examination of blood samples in this study was carried out anonymously and was approved by the local ethics review board Aerztenkammer Hamburg under number OB/V/03.

Four to fourteen milliliters of blood were collected from each patient in EDTA tubes and processed within 24 hours. The density gradient Ficoll was used for mononuclear cell enrichment: full blood was transferred to a 50 ml tube containing 30 ml HBSS (catalog no. L2045; Biochrom) and centrifuged at 400×g for 10 minutes at 4°C. Supernatant was removed by pipetting and the cell pellet was resuspended in 30 ml 1×PBS. Cell suspension was added to 20 ml Ficoll (catalog no. 17-1440-03; GE Healthcare). The mixture was spun at 400×g for 30 minutes at 4°C without acceleration and deceleration. The interface and supernatant, containing the mononuclear cells (*i.e.*, leukocytes and tumor cells), were transferred to a new 50 ml tube. The tube was filled with 1×PBS and centrifuged at 400×g for 10 minutes at 4°C. Supernatant was discarded and cell pellet was resuspended in 1 ml 1×H-Lysis buffer (catalog no. WL1000; R&D Systems) and incubated for 3 minutes with gentle shaking at room temperature. Thirty milliliters of PBS was added and sample was centrifuged again at 400×g for 10 minutes at 4°C. Supernatant was discarded and pellet was resuspended in 5 to 10 ml 1×PBS for cytospin preparation. Cell count was determined by a Neubauer counting chamber. Approximately 700.000 cells were applied to each slide.

Five milliliters of healthy volunteers’ blood were spiked with 500, 100, and 40 cell line cells. The density gradient Ficoll was used for mononuclear and spiked cells enrichment as described above. Prepared cytospins were stained with mouse IgG1 A45-B/B3-Cy3 labeled anti-human keratins 8/18/19 (Micromet) 1∶500 for 30 min and counterstained with 4',6-diamidino-2-phenylindole (DAPI) to evaluate a recovery rate of spiked cells.

Cytospins for the CTC model system and controls were obtained by spiking 500 breast cancer cell line cells into 5 ml blood from healthy volunteers followed by Ficoll gradient mononuclear cell enrichment as described above.

### Antibody detection systems and the establishment of the triple staining protocol

Cytospins of MCF7 breast cancer cells spiked into blood of healthy volunteers were stained for keratins 8/18/19 using different detection methods using anti-keratin 8/18/19 primary antibodies (Table S2). Tested methods included horseradish peroxidase-, alkaline phosphatase- and beta-galaktosidase based systems, as well as fluorescence. Slides were stained according to the protocols described in Table S2, part 1 (steps 1–6). Three single cells, positive for keratin staining, for each tested system were picked by micromanipulation and whole genome amplification (WGA) was performed. Table S2 demonstrates the whole procedure of the antibody detection including staining protocols (part 1), description of staining results (part 2), and possibility of WGA on single cells (part 3). Only two out of seven tested approaches could be used in the establishment of triple ER/K/CD45 staining: fluorescence and nitro-blue tetrazolium 5-bromo-4-chloro-3'-indolyphosphate (NBT/BCIP) based visualization provided clear staining without background and were compatible with micromanipulation and WGA of single cells. The remaining five systems demonstrated either inadequate staining and/or inhibited WGA. In detail, horseradish peroxidase substrates 3,3′-diaminobenzidine (DAB) and nickel/catachol-based DAB enhancement (HistoMark Orange from KPL) resulted in a strong background staining of leukocytes. TrueBlue substrate provided highly sensitive staining, but is stable in alcohols and water only and therefore hampering subsequent staining or single cell manipulation. New Fuchsine containing substrate of alkaline phosphatase showed high auto-fluorescence and was not compatible with WGA and therefore, was not suitable for the triple ER/K/CD45 staining establishment. Beta-galactosidase substrate X-Gal provides a clear turquoise stain without background. Unfortunately, staining results were not reproducible, and thus this method was also not suitable for the triple staining.

A single staining protocol for estrogen receptor (ER) was established on breast cancer cell line cytospins. The protocol was considered optimal when all cells were positive for ER staining on ER-positive breast cancer cell line cytospins (MCF-7 and BT474) and all cells were negative in case of ER-negative breast cancer cell lines (BT-20 and SKBR3). Figures of Data S1 show a clear positive staining for ER in MCF7 and BT474 cells (row A and C), whereas no signal could be detected in the ER negative BT-20 and MDA-231 cells (row B and D) and no background was detected in both experiments.

A double staining protocol for ER and keratin (K) was established on cytospins of MCF7 breast cancer cells. Different fluorescent visualization systems in different combinations were tested, *i.e.*, Alexa Fluor 350, 488, 546, 555, 594, Cy3, and Cy5 (data not shown). The best results were obtained in combination of ER staining visualized with Alexa Fluor 488 dye and direct keratin-Cy3 staining. Figure A of Data S2 demonstrates clear distinguishable ER (green) and K (red) staining, allowing for easy localization of signals even in all channels merged.

Next, we established a CTC model system by spiking breast cancer cell line cells in blood of healthy volunteers. This model system was used for the optimization of double ER/K staining (Figure B of Data S2) in the natural context of blood cells mimicking the clinical situation, establishment of CD45 single staining, a combination of the protocols, and the adjustment of a final triple ER/K/CD45 staining protocol.

The single staining and final triple staining protocol were both validated for unspecific binding of the primary and secondary antibodies. Rabbit normal IgG was applied instead of specific primary antibodies in order to proof specific binding of anti-ER antibodies and unspecific binding of the secondary antibodies. Figure C Figure of Data S2 shows that in absence of specific primary antibody no green staining could be detected, meaning that anti-ER and secondary antibodies demonstrate specific binding only.

### Cytospin triple staining

Slides were dried overnight at room temperature and stained according to the protocol steps described in Table 1 with 3×3 min washing in TBS between each step. After staining, slides were mounted with cover slips and Dako Glycergel Mounting Medium (Dako, C0563).

**Table 1:** The established triple staining protocol for detection and characterization of ER expression on CTC.

Step	Substance and antibodies	Concentration	Application time	Diluent	Manufacturer
1	Paraformaldehyde	0.5%	10 min	PBS	Merck, 1040051000
2	Triton X-100	0.1%	10 min	TBS	Sigma, 110K01792
3	AB serum	10%	20 min	TBS	Bio-Rad Medical Diagnostics, 805135
4	Rabbit anti-human estrogen receptor SP-1	1∶50	90 min at 37°C	TBS + 0.005% Triton X-100	Abcam, ab16660
5	Alexa Fluor 488 goat anti-rabbit	1∶500	45 min	TBS + 0.005% Triton X-100	Invitrogen, A11008
6	Mouse anti-human CD45, clone HI30	1∶400	45 min	TBS + 0.005% Triton X-100	BioLegend, 304002
7	Donkey anti-mouse IgG alkaline phosphatase labeled	1∶35	30 min	TBS + 0.005% Triton X-100	Abnova, PAB10741
8	Normal mouse IgG	1∶250	30 min	TBS + 0.005% Triton X-100	Millipore, 12-371
9	NBT/BCIP substrate	According to datasheet	15 min	TBS + 0.005% Triton X-100	Bio-Rad, 1706432
10	Mouse IgG1 A45-B/B3 – Cy3 labeled anti-human keratins 8/18/19	1∶500	30 min	TBS + 0.005% Triton X-100 + DAPI 1:500	Micromet, commercially not available

ER – estrogen receptor; CTC – circulating tumor cell; NBT/BCIP – nitro-blue tetrazolium and 5-bromo-4-chloro-3'-indolyphosphate; PBS – phosphate buffered saline; TBS – tris buffered saline.

Positive and negative staining controls were included for each procedure. Slides with MCF7 breast cancer cells spiked into blood of healthy volunteers were used as control. Positive control slide was stained according to the protocol; for negative (isotype) control, mouse normal IgG was applied instead of anti-ER antibodies.

The estimation of the ER staining intensity was based on the principle of the standard IRS scoring system [40], [41] and included following grades: no staining (negative); a weak staining (positive); a moderate staining (positive); a strong staining (positive). ER-negative cell line cells were used as standard of negative staining.

### Micromanipulation and whole genome amplification of single cells

Picking and transfer of single cells was done according to the previously established protocol by *Hannemann et al.* [42]. Briefly, each cell was picked individually by the use of a micromanipulator (the microinjector CellTram Vario and micromanipulator TransferMan NKII, Eppendorf Instruments, Hamburg, Germany), transferred in a drop of PBS onto a silanizated glass stick. The stick was immediately transferred into a 200 µl PCR reaction tube. Individual single cells in 200 µl PCR tubes can be stored at –80°C for further analysis.

Whole genome amplification was performed using the PicoPlex WGA Kit for single cells (Rubicon Genomics, R30050) according to the manufacturer’s recommendations. The WGA product was cleaned up with NucleoSEQ spin columns (Macherrey-Nagel, Germany). DNA concentration of WGA products was measured with Nanodrop 1000 (Peqlab, Erlangen, Germany). The total yield was 1.4–5.2 µg of DNA per sample.

Quality control was done by multiplex PCR as described elsewhere [43]. Briefly, four primer set were used to amplify of 100, 200, 300, and 400bp non-overlapping fragments of GAPDH gene. One hundred fifty nanogram of genomic DNA of each single cell was taken into the PCR reaction. PCR products were analyzed in a 2% agarose TAE gel. Human leukocyte DNA was used as positive control for the multiplex PCR. Negative control probe did not contain any DNA.

### *ESR1* mutation analysis

Exons 4, 6, and 8 of the gene *ESR1* (estrogen receptor 1) were amplified and sequenced. PCR was performed using AmpliTaq Gold DNA Polymerase (Applied Biosystems, N808-0240) under following conditions for each individual probe: 0.2 mM each of ATP, GTP, CTP, TTP; 2 pmol each primer; 1.25 U of Taq polymerase; 10 ng of DNA. Concentration of MgCl_2_ required was established experimentally and represented 3 mM for amplification of exons 4, 6, and 8. Oligonucleotide primers 5′-3′, used for *ESR1* mutational analysis of exon 4: forward ACATGAGAGCTGCCAACCTT, reverse CCCCACTATTTCTCCCATGA; exon 6: forward CCCTTTCATGTCTTGTGGAAG, reverse ATGCCTTTGGAGTGGGTAGA; exon 8: forward GCTCGGGTTGGCTCTAAAGT, reverse ATGCGATGAAGTAGAGCCCG.

PCR products were analyzed in 2% agarose gel. Sequencing PCR was performed using the BigDye Terminator v1.1 Cycle Sequencing Kit (Applied Biosystems, 4336774) and 40 ng of the PCR product. The sequencing was performed in a Genetic Analyzer 3130 (Applied Biosystems).

### Protocol validation on patient material

All slides, obtained after the processing of blood samples (4–20 slides per blood sample) were stained according to the established protocol and reviewed by fluorescence or light microscopy, respectively.

The most suitable approach combines chromogenic and fluorescent staining: dark blue chromogenic substrate NBT/BCIP was used for the detection of CD45, while ER and keratin were stained using Alexa 488 and Cy-3 dyes, respectively; counter staining was performed using DAPI. The staining of keratins (K8/18/19), CD45, and ER, allowed detection of CTCs in blood and simultaneous determination of the ER status of the detected CTCs. Cells were identified as CTCs if they were positive for keratin and nuclear staining and negative for CD45 staining (K+/CD45-/DAPI+). CTC status was additionally confirmed by light microscopy using the criteria: nearly round or oval shape and high nuclear/cytoplasm ratio. The CTC number variation in blood volume collected from each patient was normalized as number of CTCs detected per one milliliter of analyzed blood.

Eight CTCs from 4 patient samples (2 CTCs per patient) were picked by micromanipulation and underwent WGA as proof of principal for the feasibility of subsequent genomic analysis. Subsequent multiplex PCR of one housekeeping gene (GAPDH) was performed. Detection of expected PCR products confirms that the quality of single cell DNA after the established staining is sufficient for further genetic analysis. The *ESR1* mutation analysis of exons 4, 6, and 8 on single cells was performed.

### Statistical analyses

Statistical significance between the groups of CTC+ and CTC- patients depending on clinical disease status was calculated by Fisher’s exact test. Survival analysis of patients tested for CTCs was done using the log-rank test after dividing the patient cohorts into CTC-positive and CTC-negative groups; HRs and 95% CI were calculated using Cox proportional hazards model. Survival data is estimated from the time point of blood collection. H score of the ER staining was calculated for each CTC-positive patient and normalized in respect to the volume of analyzed blood according to the formula 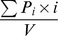, where *P_i_* - % of cells of each intensity level, *i* – intensity level (from 0 to 3), *V* – blood volume in mL. Statistical significance between the groups of patients who received endocrine therapy vs. chemotherapy at the time of blood collection was calculated by Mann-Whitney U-test.

## Results

### Spiking experiment and recovery rate

Using blood of healthy volunteers spiked with 500, 100, and 40 cell line cells we demonstrated recovery rate of 79%±4% for the density gradient Ficoll centrifugation as a method for mononuclear cell enrichment.

### CTC detection and evaluation of ER expression

We have established a triple immunostaining protocol for the simultaneous investigation of estrogen receptor (ER), keratins (K) 8/18/19, and CD45 expression on our CTC model system (blood of healthy volunteers spiked with breast cancer cell line cells) with the possibility of further single cell *ESR1* gene mutation analysis. The protocol was used for the detection and characterization of CTCs on blood samples obtained from metastatic breast cancer patients diagnosed for metastases on average 7.2 years (range: 0.5–17.0 years) after initial primary tumor resection. In total, 35 blood samples were screened by non-automated microscopy and CTCs were detected in 16 out of 35 samples (45.7%). ER staining intensity was estimated based on the following grading: no staining (negative); a weak staining (positive); a moderate staining (positive); a strong staining (positive). Samples with a weak, moderate or strong staining will be referred to as being positive for ER expression.

**Figure 1:**
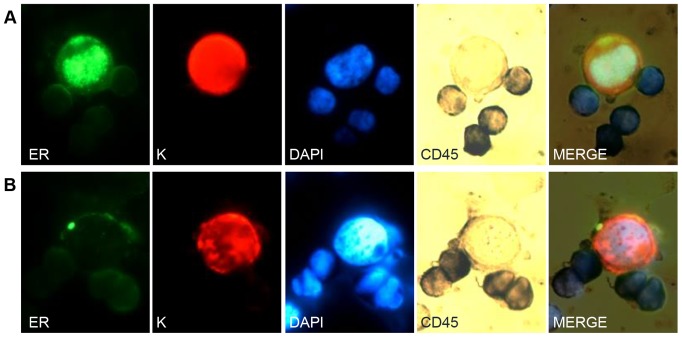
Triple immunostaining of a metastatic breast cancer patient blood sample. From left to right: estrogen receptor (ER) stained with AlexaFluor 488 (green), keratins 8/18/19 (K) stained with Cy3 (red), DAPI (fluorescent blue) for counter staining, CD45, stained with NBT/BCIP (visible dark blue), and all channels merged. Magnification x100. Row A. Images of ER-positive CTC. A cell with phenotype ER+ (green)/ K+ (red)/CD45- (dark blue)/DAPI+ (fluorescent blue) is considered to be ER-positive CTC. CTC is surrounded with leukocytes (phenotype ER-/K-/CD45+/DAPI+). Row B. Images of ER-negative CTC. A single CTC demonstrating no specific nuclear ER staining. The phenotype is ER-/K+/CD45-/DAPI+. Leukocytes present ER-/K-/CD45+/DAPI+ phenotype.

The number of detected CTCs and their ER status are presented in Table 2 (for more detailed results with grades of ER staining see Table S1). All patients had ER-positive primary tumors. ER-positive CTCs were detected in 13/16 patients totally (81.3%). Figure 1 shows a representative example of a single ER-positive CTC (Figure 1A) and a single ER-negative CTC (Figure 1B). Both cells show expression of keratins, but no expression of CD45, indicating that these are tumor cells were of epithelial origin.

**Table 2:** Number of detected CTCs and corresponding ER status.

Patient ID	Volume of analyzed blood, ml	Normalized number of detected CTCs (per 1 mL of analyzed blood)	Total number of detected CTCs (in total volume of analyzed blood)	ER-negative CTCs	ER-positive* CTCs	H score normalized (per 1 mL of analyzed blood)
069	7.2	2.78	20	17	3	3.5
072	6.3	0.16	1	1	0	0
074	7.8	0.38	3	3	0	0
076	8.7	2.53	22	10	12	12.1
241	10.9	0.73	8	5	3	8.1
243	14.0	270.0	270/1 ml	98/1 ml	172/1 ml	109
250	4.5	2.67	12	3	9	33.3
253	10.8	0.19	2	0	2	18.5
256	8.4	0.24	2	0	2	17.9
259	9.6	0.42	4	3	1	2.6
260	7.4	0.27	2	2	0	0
261	8.2	0.12	1	0	1	24.4
262	7.8	1.15	9	8	1	2.8
280	11.5	0.26	3	0	3	23.5
340	7.5	0.13	1	0	1	26.7
354	5.2	0.96	5	2	3	30.8

* ER positive group includes CTCs with weak, moderate, and strong uniform ER staining. For more detailed information see Table S1.

ER – estrogen receptor; CTC – circulating tumor cell.

Among all 16 CTC positive cases, 8 samples (50.0%) demonstrated homogeneity of ER status: 3 samples (18.7%) with ER-negative CTCs only and 5 cases (31.3%) with ER-positive CTCs only. Eight out of 16 samples (50.0%) displayed both ER-negative and ER-positive CTCs. The distribution of CTC-positive samples according to their ER status is presented in Table 3. Thus, ER-negative CTCs are present in 11/16 cases (68.7%). The average fraction of ER-negative and ER-positive CTCs in samples with mixed population was 36.8% and 63.2%, respectively.

**Table 3:** The distribution of CTC-positive samples according to their ER status and received therapy.

	CTC-positive cases		
CTC status and received therapy	all kinds of therapy, 16	women ever received ET, 14	women never received ET, 2
ER-positive only	5	4	1
ER-positive and ER-negative	8	7	1
ER-negative only	3	3	0

ER – estrogen receptor; ET – endocrine therapy; CTC – circulating tumor cell.

No significant correlation was found between the intensity and/or percentage of ER staining in the primary tumor with the number and ER status of CTCs of the same patient.

### *ESR1* mutation analysis

In subsequent experiments we investigated whether the DNA isolated from CTCs could still be used for genetic downstream analysis after triple staining and micromanipulation. The efficiency of WGA was validated with a single multiplex PCR that amplifies DNA fragments of 100, 200, 300, and 400bp from the housekeeping gene *GAPDH*. All four bands could be produced in the eight CTCs that we investigated (Data S3). Successful amplification of all these four fragments demonstrate that fragments of at least 400bp were specifically produced by the WGA for further genetic analyses [43].

Therefore, we performed mutation analysis of exons 4, 6, and 8 of the *ESR1* gene in 8 individual cells from 4 patients. Figure of Data S4 shows fragments of the high quality sequences that could be produced from all cells in the three exons. However, no mutations were found.

### CTC analysis and clinical outcome

At the time of blood sampling the disease was progressing in 15 patients out of the 16 CTC positive cases and one patient was in remission. In the CTC negative cases, 11 patients were in remission and 3 patients was in progression at the time point of the blood analysis; for 5 patients the clinical status was not evaluated at the time blood was drawn. Number of detected CTCs in respect to clinical status of the patients is presented in Table 4. Thus, the detection of CTCs was significantly associated with clinical progression of the disease (p<0.0001, two-sided Fisher’s exact test).

**Table 4:** CTC status in respect to clinical status of the patients.

	Total, 35	
Disease status	CTC positive, 16	CTC negative, 19
Progression	15	3
Remission	1	11
no data	0	5
p (Fisher’s exact test)	0.0001	

CTC – circulating tumor cell.

**Figure 2:**
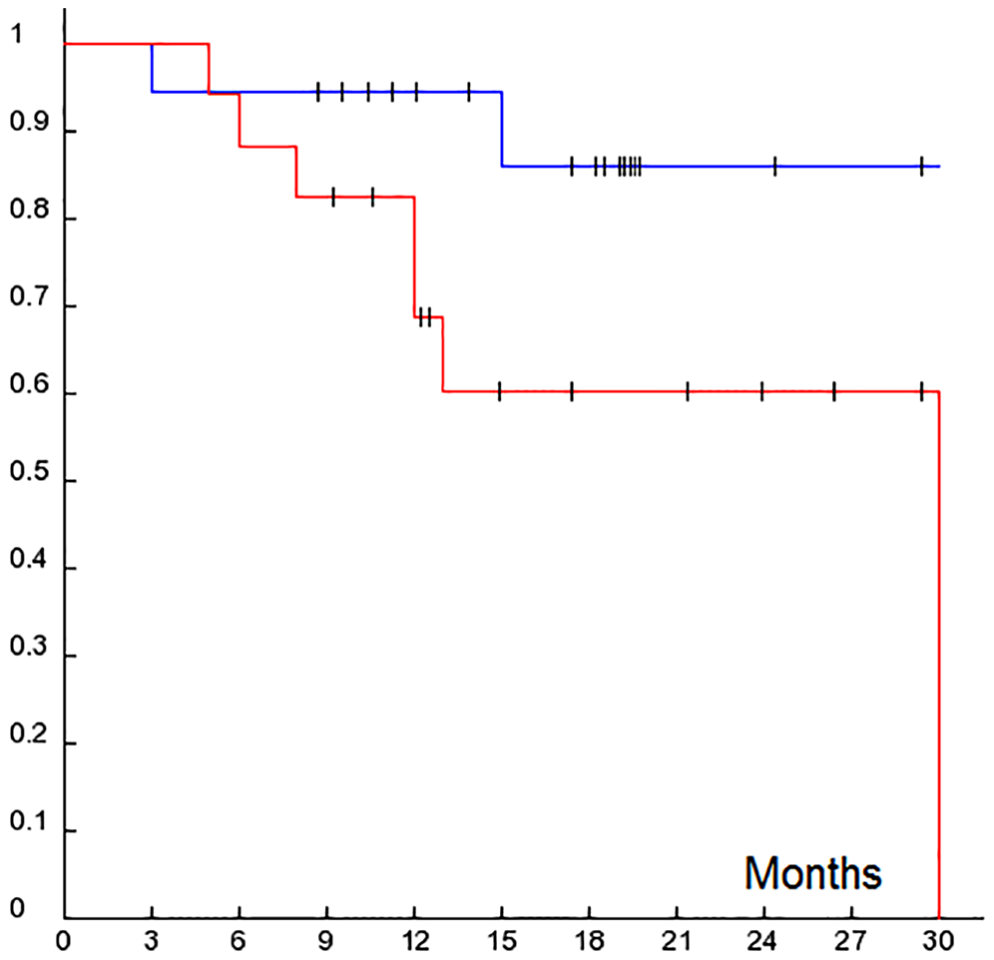
Kaplan–Meier estimate of survival function. Kaplan–Meier estimate of survival function of metastatic breast cancer patients separated on CTC-positive (red line) and CTC-negative (blue line) groups. The survival period in month of the corresponding patient. Censored patients are indicated by vertical bars (|). Statistical significance determined by log-rank test. Shorter survival correlates with presence of CTCs in blood (*P:* 0.0332, HR: 7.38, (CI = 0.84-64.09)).

Survival analysis starting from the time point of blood analysis until the end of this study (median follow up: 13.1 months, range 1–30 month), demonstrated significant correlation of CTC presence in the blood with shorter disease-free survival (p = 0.0381), as depicted by the Kaplan-Meier curves in Figure 2.

Among all 16 CTC positive cases, 14 women received endocrine therapy (87.5%), two (12.5%) did not receive endocrine therapy (Table 3). In the blood samples of women with ER-positive primary tumors that received endocrine therapy, ER-negative CTCs were found in 3/14 cases (21.47%), ER-positive CTCs in 4/14 cases (28.6%), and both ER-positive and ER-negative CTCs were detected in 7/14 patients (50.0%). Thus, presence of ER-positive CTCs in patients that received endocrine therapy was detected in 11/14 cases in total (78.6%) and ER-negative CTCs could be found in 10/14 cases (71.4%). Among the three patients in which only ER-negative CTCs were detected, two had progression of disease and therefore received chemotherapy by the time of blood analyses. One patient that developed distant metastases during endocrine therapy was switched to chemotherapy after which remission of the disease was documented.

We analyzed the normalized H score to investigate the clinical relevance of the ER intra-patient heterogeneity (Table 2 and Table S1). The groups of patients who received endocrine therapy vs. chemotherapy at the time of blood collection were compared in respect to the normalized H score for each patient (Mann-Whitney U test), but no significant correlation was found (P>0.05).

No significant correlations were found between ER status of CTCs and the following parameters: progression/remission of the disease, survival, number of detected CTCs, initial therapy, therapy by the time of blood analysis, and time to the metastases diagnosis.

## Discussion

CTCs might serve a “liquid biopsy” to investigate therapeutic targets [25]. One of the techniques often used for determining ER status of CTCs is qRT-PCR [37]–[39]; however, this approach does not allow for the investigation of intra-patient CTC heterogeneity. Therefore, in the study presented here we have investigated the expression of ER in CTCs in breast cancer patients using immunocytochemistry (ICC). With this approach, we were able to simultaneous detect and characterize CTCs with the additional possibility for downstream genetic analyses of the ER gene using whole genomic amplification (WGA).

In our study we were able to detect CTCs in 16 of 35 patient samples (45.7%), which is within the range of published reports [44]. Because EpCAM might be down regulated in tumor cells that underwent epithelial-mesenchymal transition [45], we have used an EpCAM-free detection method in order to capture as many CTCs as possible. Furthermore, we investigated ER expression in the individual keratin-positive CTCs. ER-positive CTCs were detected in 13/16 cases totally (81.3%). Primary tumors of all the patients were positive for ER with the range of ER-positive cells from 10% up to >80% of the cells. No correlation was found between the intensity of ER staining of the primary tumor and the number and/or ER status of CTCs in blood.

Others have found a concordance of ERα status between primary tumor and CTCs in metastatic breast cancer patients in 23% [37], and in 55% [39] of cases using RT-PCR approach (Table 5), which was substantially lower than our results (81.3%), obtained with ICC approach. This might be explained by the low correlation of mRNA and protein expression of ER [46]. To our knowledge, only two studies have been performed in which the authors have stained ER on single CTCs using ICC [47], [48]. Limited number of studies, based on ICC for the investigation of CTCs, might be explained by the technical challenges. These challenges had to be taken into consideration: the complications of nuclei permeabilization for antibody delivery, low level of ER presence, difficulties in unequivocal identification of CTCs in case of CD45+/K+ cells presence. A recent study by Bock and colleagues showed a higher percentage of ER-negative CTCs, however, the sample size of CTC positive metastatic breast cancer patients was relatively low (n = 5) [48]. In the study of *Nadal et al.*, in contrast to our study, only non-metastatic breast cancer patients before any systemic treatment were enrolled and volume of 30 ml blood per patient was analyzed. ER-negative CTCs were detected in 38.5% of women with ER-positive primary tumors, positive for CTCs [47].

**Table 5:** Overview of studies on ER status of CTC in metastatic breast cancer patients.

						Distribution of ER in CTCs of the patients with ER-positive primary tumors		
Assay / Approach [Ref.]	CTC enrichment	Detection of CTCs	Detection of ER	Patient cohort	CTC detection rate, %	ER-positive CTCs	ER-negative CTCs	ER-positive and ER-negative CTCs
Multiplex RT-PCR Adna Test BreastCancer [39]	anti-EpCAM and MUC1 antibodies, coupled with ferrofluidics	Multiplex PCR for mucin-1, ERBB2, actin, EPCAM	RT-PCR	42	52	6/11 (55%)	5/11 (45%)	n.a.*
Multiplex RT-PCR Adna Test BreastCancer [37]	anti-EpCAM and MUC1 antibodies, coupled with ferrofluidics	Multiplex PCR for mucin-1, ERBB2, actin, EPCAM	RT-PCR	193	45	14/62 (23%)	48/62 (77%)	n.a.*
IF [48]	Ficoll density gradient	IF for K8/18/19	IF	26	38.5	0	3/5 (60%)	2/5 (40%)
IF [present study]	Ficoll density gradient	IF for K8/18/19	IF	35	45.7	5/16 (31.3%)	3/16 (18.7%)	8/16 (50.0%)

CTC – circulating tumor cell; ER – estrogen receptor; IF – immunofluorescence; RT-PCR – real-time PCR.

* RT-PCR approach does not allow to assess intrapatient heterogeneity of ER-status of CTCs.

Because of the small number of patients investigated in our study, our follow up analysis is only of exploratory character. Nevertheless, we were able to demonstrate that the detection of CTCs in blood of metastatic breast cancer patients was significantly associated with clinical progression of the disease (p<0.0001). Although the cut-off of at least 5 CTCs per 7.5 ml of blood is considered to be the threshold of high risk of early progression in metastatic breast cancer patients using the CellSearch system [16], recent meta-analysis of *Zhang et al.*, demonstrates prognostic value of the presence of single CTCs [44]. In our study 7 patients had more than 5 CTCs in 7.5 ml of blood; nevertheless, we could demonstrate that the presence vs. absence of CTCs in blood is significantly associated with clinical progression of the disease. Moreover, it has been proposed that level of CTCs at baseline, before a new treatment for the metastatic disease starts, correlate with prognosis and outcome and might be used as independent prognostic marker of progression-free and overall survival [16]. The meta-analysis of *Zhang et al.* demonstrates that prognostic significance of CTCs’ presence does not depend on the time point of blood collection [44], which is consistent with our results where blood samples were taken during therapy.

A larger cohort with uniform treatment and longer follow-up will be required to prove the significance and clinical relevance of our findings.

Despite the considered prognostic impact of the presence of CTCs in blood, detection of CTCs in blood does not necessarily reflect the ability of CTCs to survive in the blood stream and to spread to distant organs. The survival and metastatic potential of CTCs need to be investigated.

We hypothesize that distant metastases development in women with ER-positive primary tumors during or after endocrine therapy might be related to the presence of ER-negative CTCs because these cells are most likely to be not affected by endocrine therapy.

**Figure 3:**
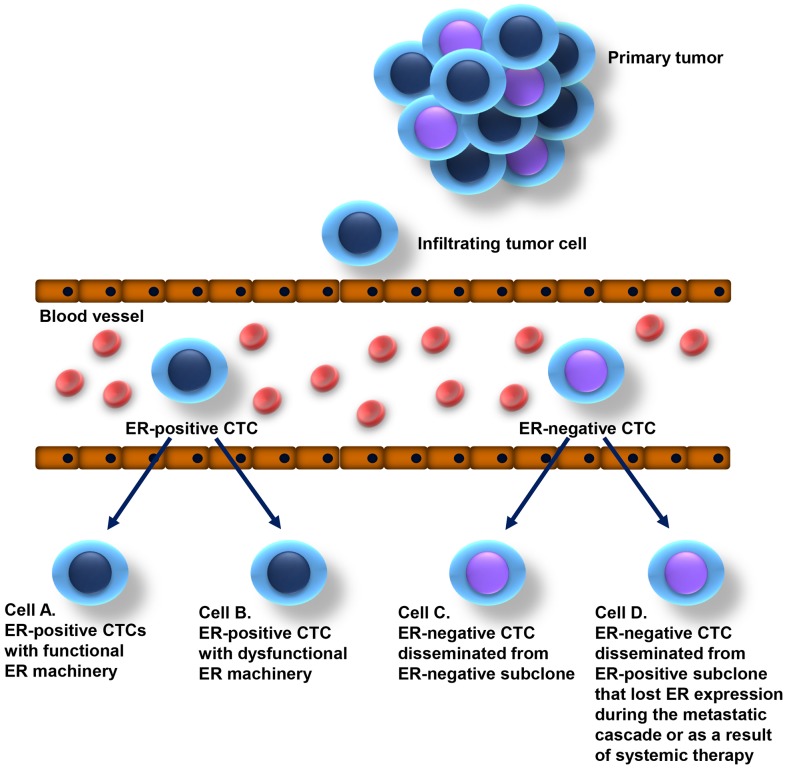
Occurrence of ER-positive and ER-negative CTCs in the peripheral blood of patients with breast carcinomas classified as ER-positive. Circulating tumor cells disseminating from an ER-positive breast tumor can be ER-positive or ER-negative. ER-positive CTCs can have normal functional ER machinery and be sensitive to endocrine therapy (cell A) or have dysfunctional ER machinery and therefore be resistant to endocrine therapy (cell B). ER-negative CTCs might disseminate from ER-negative subclones in tumors classified as ER-positive (diagnostic cut-off value: 1% of ER-stained tumor cells) (cell C) or disseminate from ER-positive subclones that lost ER expression during the metastatic cascade or as a result of systemic therapy (cell D).

Presence of ER-negative CTCs in patients with ER-positive breast cancer might be explained either by heterogeneity of primary tumor, leading to release of both ER-positive and ER-negative cells in circulation or by the switch of ER expression by genomic and/or epigenomic changes (Figure 3). It is proposed, that switching from an ER-positive to ER-negative status might be one of mechanisms to evade hormonal treatment (reviewed in [13], [33]).

We observed the presence of ER-negative CTCs in blood of women with ER-positive primary tumors during or after endocrine therapy in 10/14 cases (71.4%): 3/14 had ER-negative CTCs only (21.4%), 7/14 had ER-positive and ER-negative CTCs (50.0%). Interestingly, three of them had disease progression, receiving chemotherapy during the time of blood analyses. Further studies on larger cohorts of patients are required to determine the relevance of these findings in the context of specific endocrine therapies.

Another hypothesis, based on our observation of ER-positive CTCs in 11/14 patients after endocrine therapy, is that these cells, which are still present in blood of patients after completion of endocrine therapy, might have a dysfunctional ER pathway and, consequently, resist the hormonal ER blockade. Several mechanisms of ER-positive cells to escape anti-ER therapy have been proposed and include altered crosstalk between ER and signal transduction pathways, growth factor receptors, co-regulatory proteins of ER, and altered expression of specific microRNAs (reviewed in [33], [49]). All these mechanisms potentially lead to the loss of normal ER function and, therefore, inefficacy of anti-ER agents. Several mutations are thought to lead to the inactivation of ER and/or its ligand-independent functioning [14], [49]. Therefore, we have performed mutation analysis of the *ESR1* gene in both ER-negative and ER-positive CTCs as certain mutations hamper the protein’s function but not its expression [14]. Mutations in *ESR1* occur in approximately 1% of primary breast tumors [50], however were found in 10% of breast cancer metastases but not in the autologous primary tumors [51]. Although further investigation is required, so far we were unable to detect any mutations in the 8 single cells from 4 patients investigated in our study. However, our proof-of-principle study showed that the established immunostaining protocol is compatible with subsequent genomic analyses of CTCs, which allows for the first time a genotype-phenotype correlation at the single cell level with potential implication for future clinical studies using this information to stratify breast cancer patients to endocrine therapies, and to estimate the efficacy of endocrine therapy.

Although the intra-patient CTC heterogeneity is now a fact, a uniform scoring system for its estimation is still missing and estimation of ER expression on CTCs remains subjective. The establishment of such a system would allow for the comparison of ER heterogeneity between patients in respect to therapy as well as monitoring for intra-patient heterogeneity during/after therapy. Different approaches have been reported, nevertheless many of them base on scoring systems suggested for the estimation of IHC staining results of paraffin embedded tissue blocks. *Punnoose et al.* used a scoring system that was originally proposed by *McCarty*, but modified it for CTCs by using the sum of the positive cell percentage at each intensity level, multiplied by the weighted intensity of staining [52], [53].

Another approach was suggested by *Ligthart et al.* The authors used the mean intensity of leukocytes stained as internal threshold for each sample to quantify the intensity of HER2 expression with the use of an automated algorithm [54]. Such approach excludes subjective estimation by the investigator.

For this study, the H score system proposed by *Punnoose et al.* was used with the modification that the obtained H score was normalized to the volume of analyzed blood. This additional normalization allowed for the comparison of samples of different blood volumes. The normalized H scores for CTC-positive patients are presented in Table 2 and Table S1. We compared two groups of patients: those receiving endocrine therapy at the time of blood collection and those receiving chemotherapy, using the Mann-Whitney U-Test. It can be expected that patients who received endocrine therapy by the time of blood collection and still were in progression of the disease would demonstrate higher rates of normalized H score, than those receiving chemotherapy. In our study, the difference between the two groups was not statistically significant. Nevertheless, a larger cohort of patients is needed to study the clinical relevance of this scoring system and its impact on survival.

## Conclusion

We established a multiplex immunostaining protocol for the detection and investigation of intra-patient CTC heterogeneity, based on triple staining for keratins, ER and CD45 molecules on blood cytospins, which allows further genetic analyses of single CTCs including mutations in the *ESR1* gene. Our results demonstrate that CTCs in individual metastatic breast cancer patients with ER-positive primary tumors are frequently both ER-positive and ER-negative. ER-negative CTCs may escape ER-targeted endocrine therapy and are, therefore, a potential source of metastatic growth in breast cancer patients with ER-positive primary tumors or metastases. The investigation of CTCs for ER expression and gene status might gain future clinical utility for monitoring and optimization of breast cancer treatment.

## Supporting Information

Table S1 Patient data.
(DOCX)

Table S2 The protocol of testing, staining results and WGA compatibility of different visualization systems.
(DOCX)

Data S1 Immunofluorescent staining of estrogen receptor on breast cancer cell line cytospins using Alexa Fluor 488 dye (green) and DAPI nuclei counter staining (blue). Magnification x100. A. MCF7 breast cancer cell line cytospin demonstrating ER staining. B. BT20 breast cancer cell line cytospin demonstrating no ER staining. C. BT474 breast cancer cell line cytospin demonstrating ER staining. D. MDA-MB-231 breast cancer cell line cytospin demonstrating no ER staining.
(DOCX)

Data S2 Double immunofluorescent staining of estrogen receptor (ER), stained with AlexaFluor 488 (green) and keratins 8/18/19 (K) stained with Cy3 (red) and DAPI (blue) for nuclei counter staining. A. MCF7 breast cancer cell line cells demonstrating positivity for both ER and keratin staining. B. Cytospin of MCF7 breast cancer cell line cells spiked into blood from healthy volunteer. MCF7 single cell is positive for ER and keratin staining, leukocytes are negative for ER and keratin staining. C. Negative (isotope) control staining of MCF7 breast cancer cell line cytospin. Normal mouse IgG was applied instead of anti-ER antibodies. MCF7 cells demonstrate no green signal, but are positive for keratin staining.
(DOCX)

Data S3 Detection of multiplex PCR products of GAPDH gene in 2% agarose TAE gel. NC – negative controle (no DNA in probe), lines 1-8 – PCR products of individual single cell DNA, PC – positive controle, MM – molecularweight marker, bands top-down: 500bp, 400bp, 300bp, 200bp, 100bp. Detection of amplified 100, 200, 300, and 400bp non-overlapping fragments of GAPDH gene in probes of single cell DNA confirms appropriate quality of DNA, obtained after micromanipulation and WGA, for the downstream single cell analysis.
(DOCX)

Data S4 Sequences of the ESR1. Performed with the use of CTC DNA, which was obtained after identification and picking of the single CTC and subsequent whole genome amplification. A – fragment of the sequence of the exon 4. B – fragment of the sequence of the exon 6. C – fragment of the sequence of the exon 8.
(DOCX)
