# Supplementary material for: Heterogeneity of Estrogen Receptor Expression in Circulating Tumor Cells from Metastatic Breast Cancer Patients
Source: PLoS One. 2013 Sep 18;8(9):e75038. doi: 10.1371/journal.pone.0075038 (PMC3776726; doi:10.1371/journal.pone.0075038)
Supplement: Table S1 — Patient data. (DOCX) [file pone.0075038.s001.docx]

| **Pat ID** | **Age at FD** | **pT** | **pN** | **M** | **Grade** | **ER/IRS** | **PR/IRS** | **HER2** | **Characteristics of METs** | **Actual therapy** | **Actual disease status** | **Volume of analyzed blood, ml** | **Number of detected CTCs** | **ER status of CTCs** | **H score normalized (per 1 mL of analyzed blood)** |
| --- | --- | --- | --- | --- | --- | --- | --- | --- | --- | --- | --- | --- | --- | --- | --- |
| 069 | 29 | 2 | 1a | 0 | 2 | pos./1 | pos./6 | neg. | ER+ METs under ET, further ER+ METs under CT | CT | progress | 7.2 | 20 | 17-; 1+/-; 2+ | 3.5 |
| 072 | 67 | 2 | 3a | 1 | 2 | pos./12 | pos./12 | neg. | PMBC; progress under ET; remission after CT | CT | remission | 6.3 | 1 | 1- | 0 |
| 073 | 49 | nd | nd | 1 | nd | pos./12 | pos./12 | neg. | PMBC; progress under ET; further ER+ METs under ET | ET | remission | 8.4 | 0 | - | na |
| 074 | 44 | 2 | 0 | 0 | nd | pos./nd | pos./nd | nd | progress and further METs under ET and CT | CT | progress | 7.8 | 3 | 3- | 0 |
| 076 | nd | 2 | 3 | 1 | nd | pos./12 | neg./0 | neg. | PMBC; further METs under ET | ET | progress | 8.7 | 22 | 10-; 3+/-; 7+; 2++ | 12.1 |
| 142 | nd | 4c | 3c | 1 | 3 | pos./12 | pos./6 | pos. | PMBC | CT and Herceptin | nd | 13.2 | 0 | - | na |
| 201 | 43 | nd | nd | 1 | nd | pos./3 | neg./0 | pos. | PMBC; further METs under CT and herceptin; ET; herceptin; CT | CT | remission | 4.8 | 0 | - | na |
| 202 | 39 | nd | 0 | 0 | nd | pos./nd | nd/nd | nd | METs within the time without treatment | ET | remission | 6.7 | 0 | - | na |
| 203 | 63 | 2 | nd | 1 | nd | pos./12 | pos./nd | neg. | PMBC; progress under ET; further progress under CT; further progress under ET and CT | CT | progress | 3.8 | 0 | - | na |
| 207 | 55 | 1 | 1 | 0 | 3 | pos./nd | pos./nd | nd | ER-/HER2+ METs 8 years after ET | CT and Herceptin | remission | 7.6 | 0 | - | na |
| 213 | 45 | nd | nd | 0 | nd | pos./12 | nd/12 | neg. | relapse and METs under ET | ET | nd | 11.8 | 0 | - | na |
| 214 | 56 | 1b | 1a | 0 | 2 | pos./9 | pos./6 | neg. | ER+ METs under ET; further progress under CT | CT | nd | 10.0 | 0 | - | na |
| 215 | 37 | 1b | 0 | 0 | 2 | pos./8 | pos./4 | neg. | ER+ relapse under ET; further progress under ET | ET | remission | 9.3 | 0 | - | na |
| 222 | 52 | nd | nd | nd | 1 | pos./nd | pos./nd | pos. | further METs and progress under CT | CT and Herceptin | nd | 6.8 | 0 | - | na |
| 223 | 34 | 1c | 0 | 0 | 2 | pos./nd | pos./nd | neg. | progress of METs under ET | CT | remission | 7.5 | 0 | - | na |
| 227 | 52 | 3 | 2 | 0 | 3 | pos./9 | pos./12 | pos. | relapse and METs 1 year after ET; further METs and progress under CT and Herceptin | CT | remission | 6.9 | 0 | - | na |
| 241 | 48 | 2 | x | 1 | 2 | pos./12 | pos./8 | neg. | PMBC | ET | progress | 10.9 | 8 | 5-; 2+; 1++ | 8.1 |
| 243 | 47 | 2 | 0 | 0 | 2 | pos./2 | pos./2 | neg. | METs 4 years after ET; further ER+ METs under ET | ET | progress | 14.0 | 270/1 ml | 98-; 57+/-;  108+; 7++ | 109 |
| 247 | 57 | nd | nd | 0 | nd | pos./nd | nd/nd | nd | ER+ METs within the time without treatment | ET | nd | 6.6 | 0 | - | na |
| 249 | 49 | 4 | + | 1 | 2 | pos./12 | pos./9 | neg. | PMBC | ET | remission | 4.6 | 0 | - | na |
| 250 | 34 | 2 | + | 1 | 2 | pos./6 | pos./4 | pos. | PMBC; progress under CT and herceptin | CT | progress | 4.5 | 12 | 3-; 9+ | 33.3 |
| 253 | 24 | 2 | 0 | 0 | 2 | pos./nd | pos./nd | neg. | multiple METs under ET; further progress under CT and ET | ET | progress | 10.8 | 2 | 2+ | 18.5 |
| 255 | nd | 2-3 | 3c | 1 | nd | pos./12 | pos./12 | neg. | PMBC | CT | remission | 11.0 | 0 | - | na |
| 256 | 45 | 4 | 3 | 1 | 2 | pos./4 | pos./12 | neg. | PMBC | CT | progress | 8.4 | 2 | 1+/-; 1+ | 17.9 |
| 259 | 39 | 1b | 0 | 0 | 2 | pos./6 | pos./nd | neg. | ER+ relapse and METs 2 years after ET | CT | progress | 9.6 | 4 | 3-; 1+/- | 2.6 |
| 260 | 50 | nd | nd | 1 | 3 | pos./4 | pos./6 | neg. | PMBC; further progress under CT and ET; | CT | progress | 7.4 | 2 | 2- | 0 |
| 261 | 47 | nd | nd | 0 | nd | pos./nd | nd/nd | neg. | relapse 3 month after ET; METs 4 years after ET; further progress under ET and CT | CT | progress | 8.2 | 1 | 1+ | 24.4 |
| 262 | 67 | 4d | 1 | 1 | 2 | pos./12 | pos./12 | neg. | PMBC; further progress under ET | ET | progress | 7.8 | 9 | 8-; 1+ | 2.8 |
| 263 | 72 | nd | nd | 1 | nd | pos./nd | pos./nd | neg. | PMBC; further progress under ET | ET | progress | 4.8 | 0 | - | na |
| 266 | 56 | 3 | 1 | 0 | 3 | pos./nd | pos./nd | nd | METs 4 years after ET | CT | progress | 4.8 | 0 | - | na |
| 280 | 32 | 2 | 0 | 0 | 3 | pos./12 | pos./nd | neg. | ER+ METs within the time without treatment; further progress under ET and CT | CT | progress | 11.5 | 3 | 1+; 2++ | 23.5 |
| 340 | 45 | 2 | 0 | 1 | 1 | pos./nd | neg./0 | neg. | PMBC; further progress under ET and CT | CT | progress | 7.5 | 1 | 1+ | 26.7 |
| 354 | 59 | 2 | 1 | 0 | 3 | pos./6 | pos./4 | neg. | METs after CT and ET; further progress under CT | CT | progress | 5.2 | 5 | 2-; 1+; 2++ | 30.8 |
| 359 | 72 | 2 | 3a | 1 | 3 | pos./6 | pos./6 | pos. | PMBC | ET | remission | 6.0 | 0 | - | na |
| 363 | 57 | 2 | 0 | 0 | 3 | pos./12 | pos./6 | neg. | METs after CT | CT | remission | 6.7 | 0 | - | na |

CT – chemotherapy;

CTC – circulating tumor cell;

ER – estrogen receptor;

ERBB2 - receptor tyrosine-protein kinase erbB-2;

ET – endocrine therapy;

FD – first diagnosis;

Grade – histologic grade, given according to Nottingham score system;

IRS – german scoring system, reflecting nuclear staining intencity and percentage of stained cells in breast cancer sample. The scale 0-12 represents all grades from negative staining (score 0) to strong positive in more than 80% of tumor cells (score 12);

MET – metastases;

na – not available;

nd – no data;

neg. – negative status;

Parameters T, N, M – are given according standard TNM classification;

PMBC – primary metastatic breast cancer;

pos. – positive status;

PR – progesterone receptor;

*ER status of CTCs was estimated by ER staining intensity and graded as following:

“-“ – no staining;

“+/-“ – a weak staining;

“+” – a moderate staining;

“++” – a strong staining.
