# Supplementary material for: Heterogeneity of Estrogen Receptor Expression in Circulating Tumor Cells from Metastatic Breast Cancer Patients
Source: PLoS One. 2013 Sep 18;8(9):e75038. doi: 10.1371/journal.pone.0075038 (PMC3776726; doi:10.1371/journal.pone.0075038)
Supplement: Table S2 — The protocol of testing, staining results and WGA compatibility of different visualization systems. (DOCX) [file pone.0075038.s002.docx]

| Visualization system | | Horseradish peroxidase-based systems | | | Alklaline phosphatase-based systems | | Beta-galactosidase-based system | Fluorescence-based system |
| --- | --- | --- | --- | --- | --- | --- | --- | --- |
| Substrate/conjugate | | HistoMark ORANGE Substrate (KPL, 54-74-00) | DAB+ Substrate (Dako, K3468) | TrueBlue Substrate (KPL, 71-00-67) | New Fuchsine Substrate | NBT/BCIP Substrate (Bio-Rad, 1706432) | HistoMark X-Gal Substrate (KPL, 54-13-00) | Cy3-conjugate |
| Part 1. Staining procedure | Step 1. Fixation | 4% paraformaldehyde in PBS, 10 min | | | | | | |
|  | Step 2. Blocking | 10% AB serum in PBS (Bio-Rad Medical Diagnostics, 805135), 20 min | | | | | | |
|  | Step 2a. Additional blocking | indigenous peroxidase blocking with Blocking Solution Concentrate, 1:10, 1 min (KPL, 71-00-10) | | | - | | avidin block: avidin in PBS 1.5 μM 20 min, Calbiochem,189725;  biotin block: biotin in PBS 2 mM 20 min, Applichem, A0969 | - |
|  | Step 3. Primary antibodies application | Mouse anti-human keratin 8/18/19 monoclonal antibodies A45-B/B3 19  (Micromet, R002A) in 10% AB serum, 45 min | | | | | | Mouse IgG1 A45-B/B3 – Cy3 labeled anti-human keratins 8/18/19 in 10% AB serum, 30 min (Micromet) |
|  | Step 4a. Secondary antibodies application | - | | | - | | polyclonal rabbit anti-mouse IgG biotinylated, 1:100, 45 min (Dako, E046401) | - |
|  | Step 4b. Enzyme-conjugate application | ZytoChem Plus HRP One-Step Polymer anti-mouse (ZUC050-006), 30 min | | | Donkey anti-mouse IgG alkaline phosphatase labeled, 1:35 in 10% AB serum, 30 min (Abnova, PAB10741) | | Beta-galactosidase avidin D, 1:30, 45 min (Vector Laboratories, A-2300) | - |
|  | Step 5. | substrate solution (prepared according to the datasheet), 10 min in darkness | substrate solution (prepared according to the datasheet), 20 min in darkness | peroxidase TrueBlue substrate, 10 min | homemade New Fuchsine substrate, 20 min | NBT/BCIP substrate, prepared according to the datasheet, 15 min (Bio-Rad,1706432) | X-Gal substrate (prepared according to the datasheet), 30 min at 37°C | - |
|  | Step 6. Counterstaining | DAPI 1:500, 2 min | | | | | | |
| Part 2. Description of staining result. | | high background staining of leukocytes | high background staining of leukocytes | precipitate is fading in PBS/TBS and aqueous mounting media | fluoresce | appropriate | not reproducible results | appropriate |
| Part 3. Possibility of whole genome amplification | | possible | possible | possible | inhibited | possible | not performed due to irrepro-ducibility of staining | possible |

Washing 3x 3 min in TBS was performed between each step.
