# Supplementary figures and images for: Heterogeneity of Estrogen Receptor Expression in Circulating Tumor Cells from Metastatic Breast Cancer Patients
Source: PLoS One. 2013 Sep 18;8(9):e75038. doi: 10.1371/journal.pone.0075038 (PMC3776726; doi:10.1371/journal.pone.0075038)

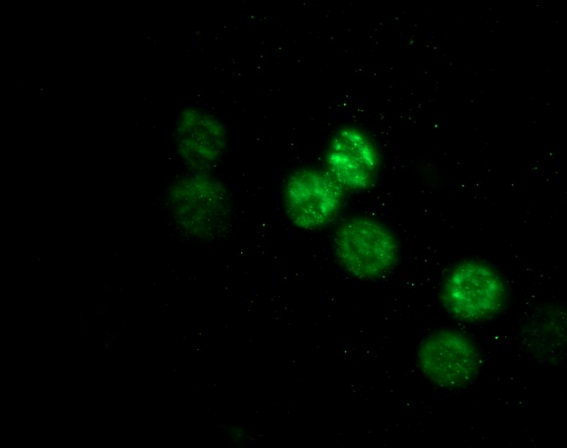

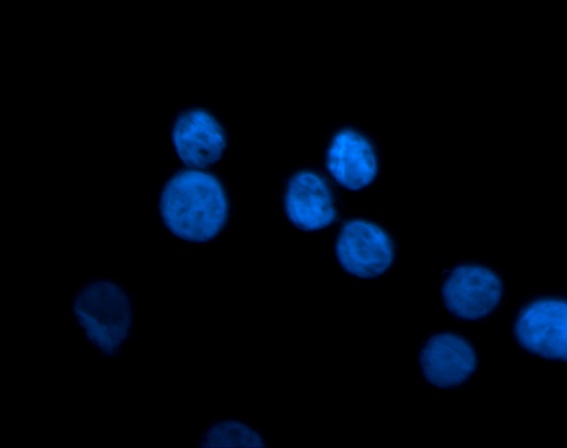


**A**

**B**

**C**

**D**

**ER DAPI MERGE**


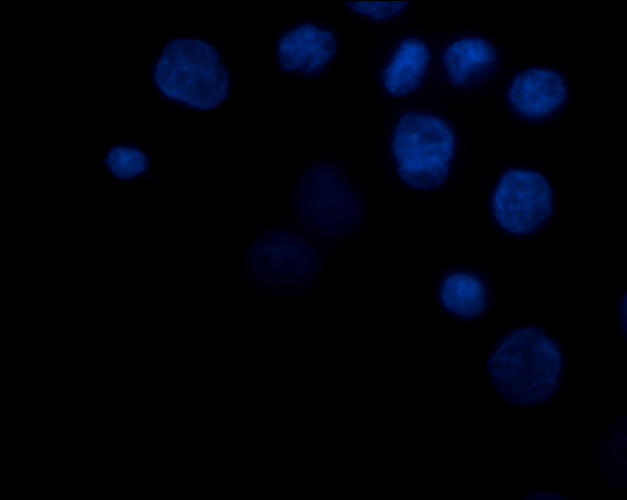

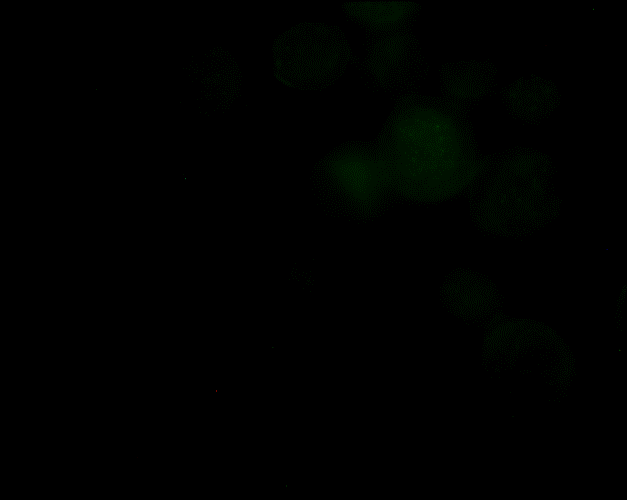


**ER DAPI MERGE**


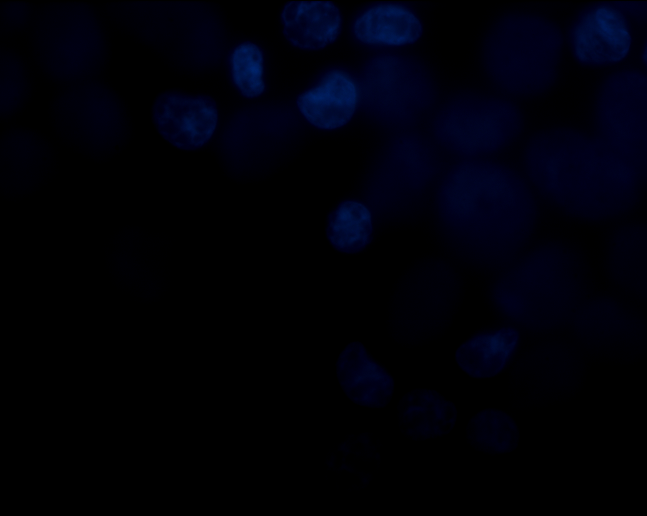

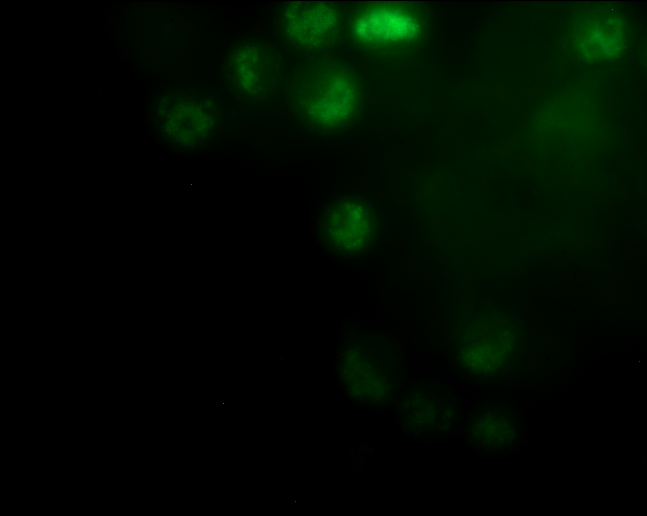


**ER DAPI MERGE**


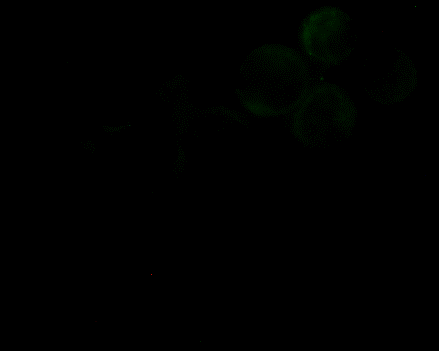


**ER DAPI MERGE**

Supplement: Data S1 — Immunofluorescent staining of estrogen receptor on breast cancer cell line cytospins using Alexa Fluor 488 dye (green) and DAPI nuclei counter staining (blue). Magnification x100. A. MCF7 breast cancer cell line cytospin demonstrating ER staining. B. BT20 breast cancer cell line cytospin demonstrating no ER staining. C. BT474 breast cancer cell line cytospin demonstrating ER staining. D. MDA-MB-231 breast cancer cell line cytospin demonstrating no ER staining. (DOCX) [file pone.0075038.s003.docx]

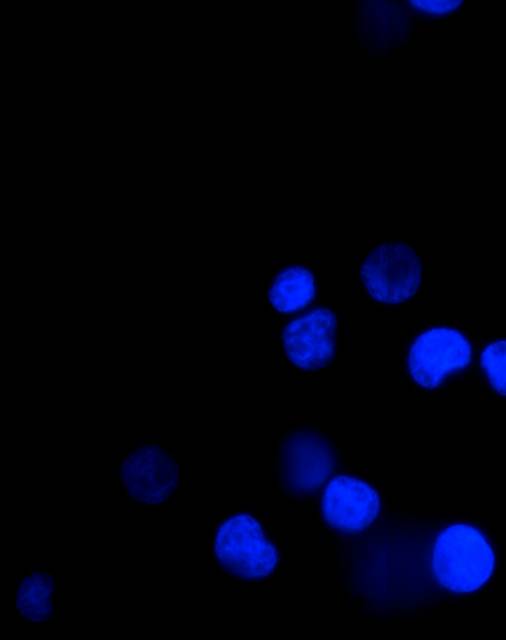

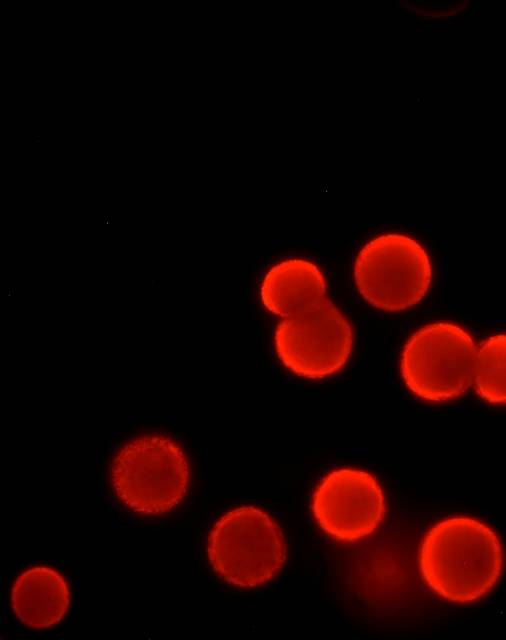

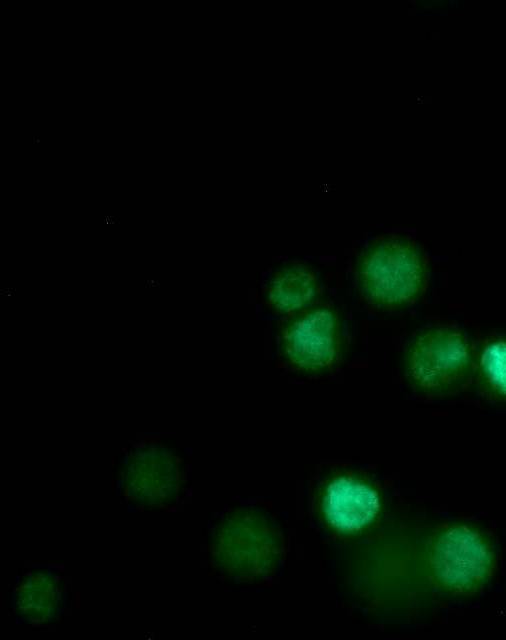


**A**

**B**

**C**

**x40 x40 x40 x40**

**ER K DAPI MERGE**


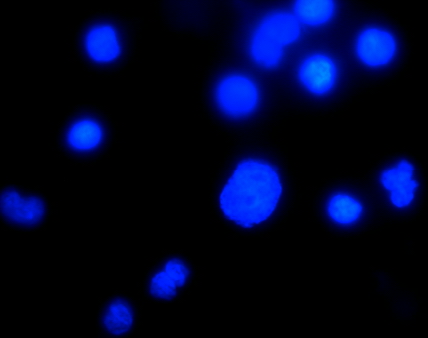

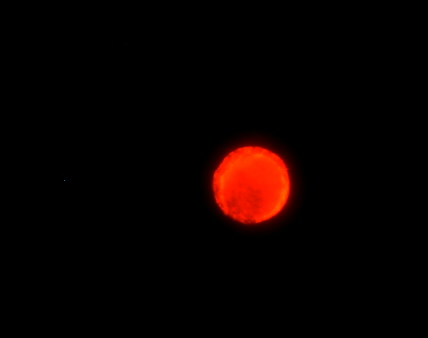

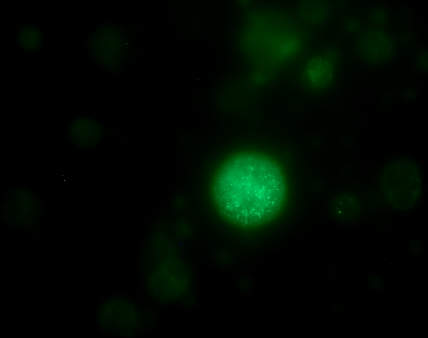


**x100 x100 x100 x100**

**ER K DAPI MERGE**


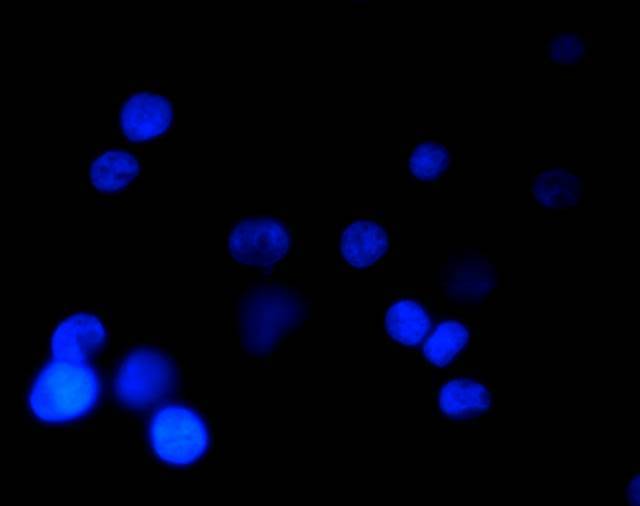

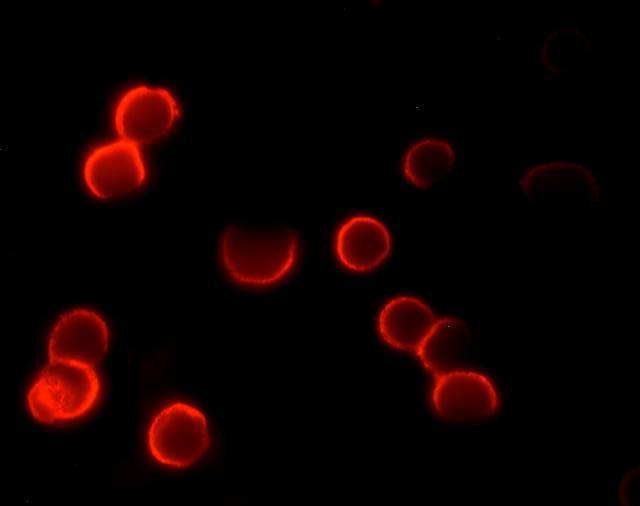

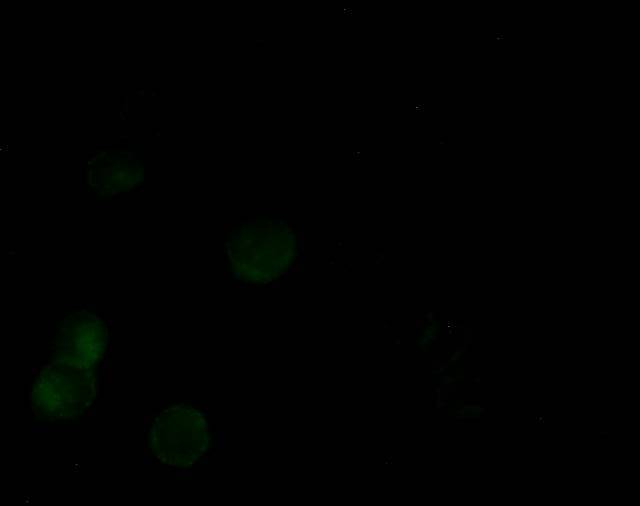


**x40 x40 x40 x40**

**ER K DAPI MERGE**

Supplement: Data S2 — Double immunofluorescent staining of estrogen receptor (ER), stained with AlexaFluor 488 (green) and keratins 8/18/19 (K) stained with Cy3 (red) and DAPI (blue) for nuclei counter staining. A. MCF7 breast cancer cell line cells demonstrating positivity for both ER and keratin staining. B. Cytospin of MCF7 breast cancer cell line cells spiked into blood from healthy volunteer. MCF7 single cell is positive for ER and keratin staining, leukocytes are negative for ER and keratin staining. C. Negative (isotope) control staining of MCF7 breast cancer cell line cytospin. Normal mouse IgG was applied instead of anti-ER antibodies. MCF7 cells demonstrate no green signal, but are positive for keratin staining. (DOCX) [file pone.0075038.s004.docx]

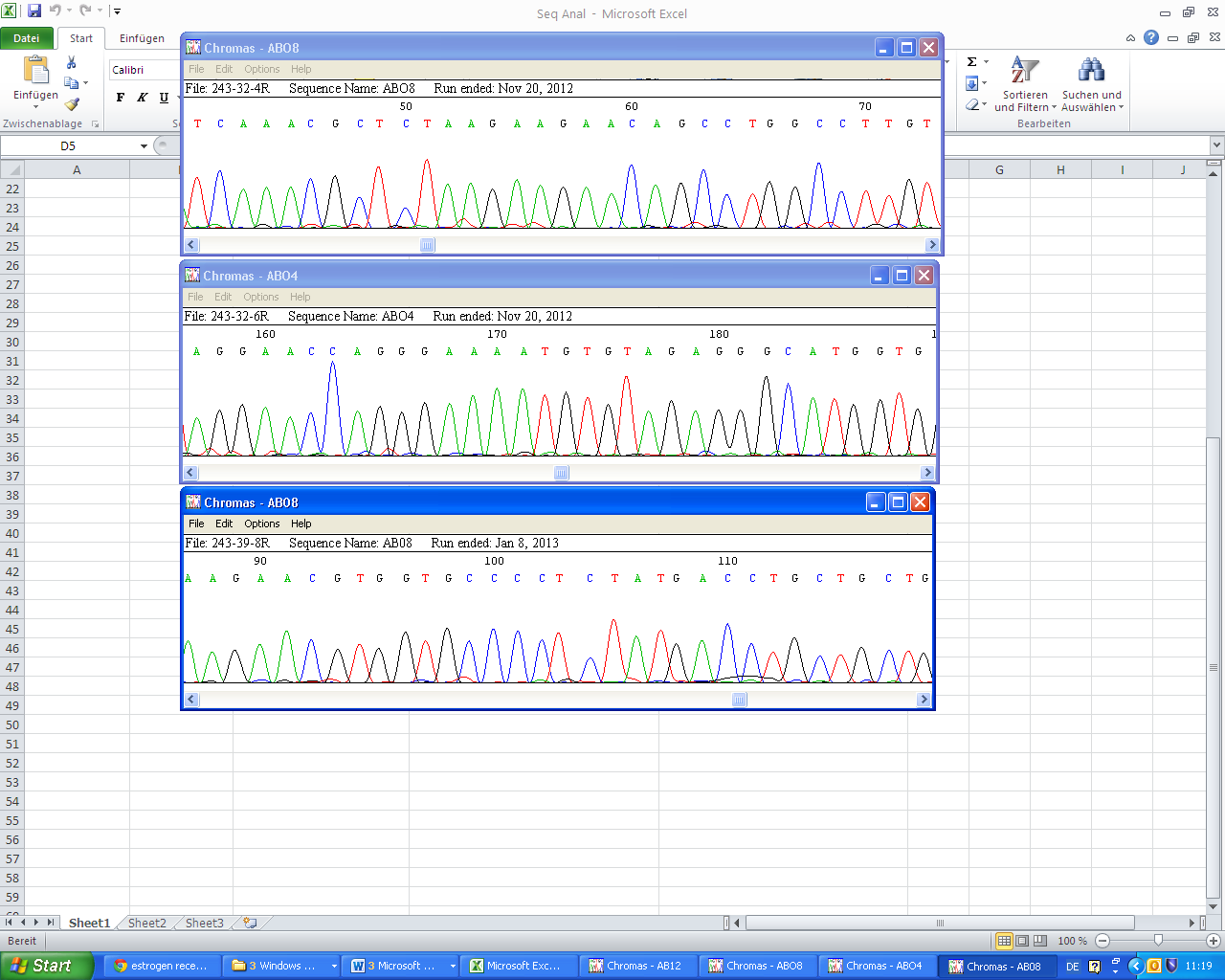


A

B

C

Supplement: Data S4 — Sequences of the ESR1. Performed with the use of CTC DNA, which was obtained after identification and picking of the single CTC and subsequent whole genome amplification. A – fragment of the sequence of the exon 4. B – fragment of the sequence of the exon 6. C – fragment of the sequence of the exon 8. (DOCX) [file pone.0075038.s006.docx]
