# Supplementary material for: Heterogeneity of Estrogen Receptor Expression in Circulating Tumor Cells from Metastatic Breast Cancer Patients
Source: PLoS One. 2013 Sep 18;8(9):e75038. doi: 10.1371/journal.pone.0075038 (PMC3776726; doi:10.1371/journal.pone.0075038)
Supplement: Data S3 — Detection of multiplex PCR products of GAPDH gene in 2% agarose TAE gel. NC – negative controle (no DNA in probe), lines 1-8 – PCR products of individual single cell DNA, PC – positive controle, MM – molecularweight marker, bands top-down: 500bp, 400bp, 300bp, 200bp, 100bp. Detection of amplified 100, 200, 300, and 400bp non-overlapping fragments of GAPDH gene in probes of single cell DNA confirms appropriate quality of DNA, obtained after micromanipulation and WGA, for the downstream single cell analysis. (DOCX) [file pone.0075038.s005.docx]

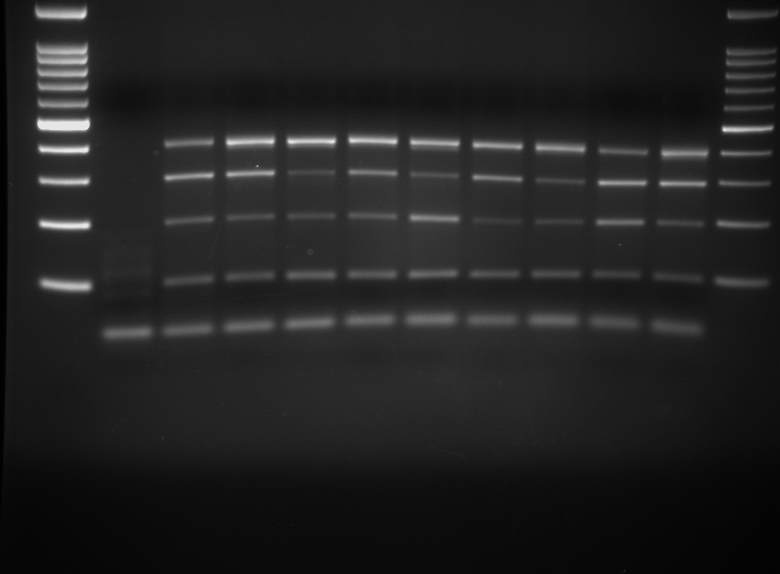


NC 1 2 3 4 5 6 7 8 PC MM

NC – negative controle (no DNA in probe), lines 1-8 – PCR products of individual single cell DNA, PC – positive controle, MM – molecularweight marker, bands top-down: 500bp, 400bp, 300bp, 200bp, 100bp. Detection of amplified 100, 200, 300, and 400bp non-overlapping fragments of GAPDH gene in probes of single cell DNA confirms appropriate quality of DNA, obtained after micromanipulation and WGA, for the downstream single cell analysis.
